# Designing Ethical Social Robots—A Longitudinal Field Study With Older Adults

**DOI:** 10.3389/frobt.2020.00001

**Published:** 2020-01-24

**Authors:** Anouk van Maris, Nancy Zook, Praminda Caleb-Solly, Matthew Studley, Alan Winfield, Sanja Dogramadzi

**Affiliations:** ^1^Bristol Robotics Laboratory, University of the West of England, Bristol, United Kingdom; ^2^Department of Health and Social Sciences, University of the West of England, Bristol, United Kingdom; ^3^Department of Automatic Control and Systems Engineering, The University of Sheffield, Sheffield, United Kingdom

**Keywords:** social robots, older adults, longitudinal study, ethics, deception, attachment

## Abstract

Emotional deception and emotional attachment are regarded as ethical concerns in human-robot interaction. Considering these concerns is essential, particularly as little is known about longitudinal effects of interactions with social robots. We ran a longitudinal user study with older adults in two retirement villages, where people interacted with a robot in a didactic setting for eight sessions over a period of 4 weeks. The robot would show either non-emotive or emotive behavior during these interactions in order to investigate emotional deception. Questionnaires were given to investigate participants' acceptance of the robot, perception of the social interactions with the robot and attachment to the robot. Results show that the robot's behavior did not seem to influence participants' acceptance of the robot, perception of the interaction or attachment to the robot. Time did not appear to influence participants' level of attachment to the robot, which ranged from low to medium. The perceived ease of using the robot significantly increased over time. These findings indicate that a robot showing emotions—and perhaps resulting in users being deceived—in a didactic setting may not by default negatively influence participants' acceptance and perception of the robot, and that older adults may not become distressed if the robot would break or be taken away from them, as attachment to the robot in this didactic setting was not high. However, more research is required as there may be other factors influencing these ethical concerns, and support through other measurements than questionnaires is required to be able to draw conclusions regarding these concerns.

## 1. Introduction

Awareness of, and a growing interest in, ethical considerations for the development of social robots is increasing due to the predicted increasing likelihood of robots being a part of our everyday lives in the future (Malle et al., 2015; Esposito et al., 2016; Li et al., 2019). This is evident through the emergence of relatively new conferences like the International Conference on Robot Ethics and Standards^1^, and new ethical standards in robotics and AI (Winfield, 2019). Socially assistive robots can provide psycho-social, physical and/or cognitive support while interacting with their users (Robinson et al., 2014). Therefore, potential ethical concerns of prolonged use of social assistive robots needs to be considered while these systems are still being developed. This will help to ensure that appropriate safeguards are considered and built into systems as an integral part of their design. In addition, this will facilitate clear guidelines and regulations for safe deployment. One area for investigation that has been identified is the use of emotional expression in the robot, which can lead to emotional deception (Sharkey and Sharkey, 2012). Emotional deception could occur when the user believes that the robot really experiences these emotions, leading to unrealistic expectations that can possibly result in the user prioritizing the robot's well-being over other people's or their own well-being, as well as over-relying on the robot as a social assistant without exerting one's own critical judgment (Fulmer et al., 2009). Another ethical concern is the possible development of emotional attachment to the robot (Sullins, 2012), which may cause distress in the user when the robot breaks or is taken away. Whilst these issues are important to consider in all human-robot interactions, the current study focuses on self-reported healthy older adults. This group was selected due to the emergence of social robots as a way to support caregivers and care homes as they meet a growing demand for care for the aging population (Unies, 2015). Safe and responsible introduction of social robots to this target group is essential, as a potential lack of knowledge of and experience with new technologies may lead to situations that potentially affect psychological and/or physical safety (Borenstein et al., 2017). Moreover, the step between utilizing robots for cognitively healthy older adults to vulnerable older adults that suffer from e.g., dementia is small, and baseline requirements found through studies with healthy older adults are essential to ensure that it is ethically safe for vulnerable older adults to interact with the robot.

Frennert and Östlund described several matters of concern that arise during the development of social robots for older adults (Frennert and Östlund, 2014). Some of these entail the role that the robot will play in the older adults' lives, factors that can influence social robot acceptance, methodology used in robotic research and ethical implications. These matters were addressed in this study. A specific role for the robot was determined and communicated to the participants, namely that of a didactic learning companion. Factors that may influence acceptance were investigated, and whether these could have ethical consequences. We addressed methodology concerns and even though the number of participants is still low, we did use a comparison group (Bemelmans et al., 2012) and ran the study in a naturalistic setting. As the goal is to ensure that social interactions are ethically safe and acceptable, the concern that social interactions are driven by technological determinism has been addressed as well.

The aims of this study are to establish whether the ethical concerns of emotional deception and emotional attachment that have been established in the literature are reflected in practice. More specifically, this study investigates whether older adults are emotionally deceived by a robot when it shows emotional expressions during didactic interaction in a naturalistic setting, and whether they will become emotionally attached to the robot over time. Suggestions for how the social robot could be adapted to address ethical and acceptability concerns are considered. As no similar study has been previously conducted in the literature, this work could provide useful insights into conducting longitudinal field studies and lay the foundation for future work with vulnerable populations of older adults, such as those with dementia.

The following hypotheses are investigated in this study: It is expected that effects of emotional deception will be minimal, as the level of deception was designed to be low. Furthermore, any effects that occur will decrease over time, once participants become familiar with the displayed emotions of the robot. Additionally, it is hypothesized that emotional attachment will initially be low but increase over time, and that attachment will be higher for participants interacting with the emotive robot, as the display of emotions by the robot will increase people's perception of the robot being a social entity.

## 2. Social Robots and Human-Robot Interaction

One issue relating to human-robot interaction and social robotics relates to the lack of a common definition for a social robot. For example, Dautenhahn and Billard state that a social robot is an embodied agent that is part of a society of robots and/or humans (Dautenhahn and Billard, 1999). This statement is followed by the notion that these agents can recognize one another, join a social interaction, and learn from each other. An alternative definition is provided by Fong, who describe a social robot as an agent for which interaction is important (Fong et al., 2003). This lack of consensus regarding a definition presents challenges when developing a framework for the investigation of social robots, as it is difficult to ensure all parties involved envisage the same outcome without a common definition to refer to. Combining research by Breazeal and Fong, we can distinguish seven different classes of social robots: socially evocative, social interface, socially receptive, sociable, socially situated, socially embedded and socially intelligent (Fong et al., 2003; Breazeal, 2004). In this research, we will focus on robots from the first two categories, socially evocative and social interface, since these require little social cognition and will be easier to use in real-world settings in less time, but will, due to the small amount of social cognition required, raise several possible ethical concerns.

### 2.1. Ethical Concerns in HRI

The population of older adults is growing, and the demand for care is growing with them (Unies, 2015). However, the capacity to supply this call for care is limited. This is one of the reasons why research in robotics is so attractive (Sparrow and Sparrow, 2006). Use of social robots by older adults will require ongoing care and health education. The fact that social robots are not designed to be influenced by an emotional state, nor judge people (Breazeal, 2011) might make them less stigmatizing to use in this context (Breazeal, 2011).

Several ethical implications of using robots for older adults have been established in the literature. Example implications are reduced human contact, loss of control, loss of personal liberty, loss of privacy, matters regarding responsibility, infantilization, emotional deception and emotional attachment (Sharkey and Sharkey, 2012; Sullins, 2012; Kolling et al., 2013). Even though these are all valid concerns, there are counterarguments against several of these as well. Some of these counterarguments have been raised by Sharkey and Sharkey themselves (Sharkey and Sharkey, 2012). For example, the use of social robots may reduce people's contact with others, but it can also reduce isolation and increase conversation opportunities both with the robot and other users (Sharkey and Sharkey, 2012). Other ethical issues are loss of control and loss of personal liberty, but robots can also give older people the opportunity to self-manage their well-being and the ability to reduce risks (Callén et al., 2009). Privacy issues are equally important, but do not apply solely to social robotics and are being investigated for many other technologies deployed in human environment and, therefore, will not be discussed in this paper, as well as matters regarding responsibility, which are being researched through e.g., autonomous cars. Encouraging people to interact with robots, that can sometimes have a toy like appearance, might give them the feeling they are being infantilized. However, this can be addressed by taking into account the aesthetics of the robot and including the older adults in the development process, which have been identified as a matters of concern when developing robots for older adults (Frennert and Östlund, 2014). This leaves two ethical concerns: emotional deception and emotional attachment. These two concerns were investigated in more depth in this study.

#### 2.1.1. Emotional Deception

Deception occurs when false information is communicated to benefit the communicator (Arkin et al., 2012); it implies that an agent acts in a way that it induces a false belief in another agent (Hyman, 1989). This can also mean that no information is communicated at all (Dragan et al., 2015). Deception can be approached through different perspectives like philosophy, economics, and biology (Shim and Arkin, 2013). Both the perspective of philosophy and biology discuss the division of deception into either unintentional or intentional (Dragan et al., 2015). Unintentional deception takes place when some feature of the (unintentional) deceiver evokes unforeseen expectations in the agent being deceived. Intentional deception takes place when the deceiver is aware that these features will raise false expectations in the agent being deceived. This distinction suggests that emotional deception is not a binary materialization, but a spectrum with different gradations (Winkle and Van Maris, 2019).

Deception is generally perceived as bad. However, this is not necessarily the case. An example where deception benefits the agent being deceived is the use of placebo-effect. This beneficial form of deception is called benevolent deception (Adar et al., 2013). Benevolent deception has always been part of medical care (Jackson, 1991), and may even be required to act morally (Arkin et al., 2012).

Deception is created when robots are used in assistive settings (Sharkey and Sharkey, 2011), since the robot's social behavior often does not correspond with its actual capabilities. This could be a risk, since users may perceive robots differently than intended and raise expectations that cannot be met by the robot. For a robot to be able to successfully perform a deceptive action, it requires specific knowledge about the person that it intends to deceive (Wagner and Arkin, 2011). Also, the robot should convey its intentions and have a theory of mind for the person being deceived to be able to manipulate their beliefs (Dragan et al., 2015). However, an area that is often not discussed is the fact that a robot can also unintentionally deceive through its appearance and/or behavior.

When objects, in this case the robot, provide social support, interactions may be more effective (Kidd et al., 2006). More effective interactions will lead to a better interaction quality and improvement of quality of life, which is the main reason why robots are used in care for older adults. Providing a robot with emotive behavior is a way to improve its capability to communicate with a person (Kirby et al., 2010). People are capable of recognizing facial expressions in robots (Kirby et al., 2010), and perceive the emotion they recognize themselves as well.

Displays of robot-human affection would be an appearance of affection from the robot toward the human, as real affection requires emotions, which are difficult to implement in robots (Weijers, 2013). Being able to convey emotions is a requirement for a successful companion robot (Breazeal and Scassellati, 1999). However, one might argue this is a form of deception, as the robot does not actually experience emotions. Especially, as emotional deception is stated as the misrepresentation of one's emotional state (Fulmer et al., 2009), and the robot provides incorrect information about its internal state when displaying emotive behavior. As the perception of emotional deception is a subjective response it is measured indirectly through other variables in this study. Older people may benefit from emotive robot behavior, as receiving little affection can have negative consequences for people that are feeling lonely like cardiovascular function (Cacioppo and Patrick, 2008). Whether emotional deception by a robot is benevolent and thus ethically acceptable, especially when interacting with vulnerable users, has to be researched more thoroughly, which was one of the aims of this study. The results provided insights in effects of emotional deception in a didactic setting, which have not been investigated yet.

#### 2.1.2. Emotional Attachment

Attachment can be described as the sum of cohesion episodes that a person has made with other persons or objects (Huber et al., 2016). A cohesion episode entails joint experiences with these other people or objects, in which cohesion factors are present. Cohesion factors can be defined as shared factors like values and preferences, charisma factors like attractiveness and sympathies, personal factors like expressed openness and social factors like non-situation-specific reciprocity).

Research on attachment and robots has either focused on robots showing attachment to the user or eliciting attachment in the user through its behavior (e.g., Hiolle et al., 2009, 2012). It is possible to become attached to a robot, as people are capable of becoming attached to objects (Keefer et al., 2012). Scheutz has highlighted that there is very little needed for people to become attached to robots, even if these robots do not show behavior that elicits attachment (Scheutz, 2011). Therefore, it is particularly important to explore attachment in socially assistive robots interacting with older adults. There are four attachment styles that distinguish how easily a person becomes attached to someone or something: secure attachment, fearful attachment, preoccupied attachment and dismissive attachment (Brennan et al., 1998). Since social robots (and other assistive technologies) become more advanced, the likeliness of users forming attachment-like bonds to them increases (Collins et al., 2013). Opinions on whether it is acceptable for a robot to elicit attachment in its users are divided. On the one hand, eliciting attachment will support the process and goals of its use (Coeckelbergh et al., 2016). Some even say that eliciting attachment in its users is a necessity for the robot to be fully effective in a care providing context (Birnbaum et al., 2016). However, once users have become attached to the robot, taking it away may cause emotional distress (Sharkey and Sharkey, 2010; Coeckelbergh et al., 2016).

Emotional deception and emotional attachment have been raised as ethical concerns in the literature. However, this has never been investigated in practice, which was one aim of this study. Whether these concerns are reflected in practice was investigated through a longitudinal human-robot interaction study, where people's acceptance of the robot, perception of the social interaction and attachment to the robot were measured over time. The robot's behavior was manipulated to investigate emotional deception. This study investigated how emotional deception and emotional attachment may relate to acceptance of the robot and perception of the social interaction, as these will be indicators for the future development of ethically safe socially assistive robots.

## 3. Methods

### 3.1. Overview

The aim of this study was to investigate emotional deception and emotional attachment. Participants' responses to a robot displaying either emotive or non-emotive behavior, and their level of attachment to the robot over time were investigated. Questionnaires were administered several times during the experiment to study participants' attachment to the robot, their acceptance of the robot, and their perception of the social interactions with the robot. Most participants interacted with the emotive and the non-emotive robot, except for a small control group that interacted with the non-emotive robot only. Other data regarding the participants' affect, physiological state and behavior were also measured using sensors and video recordings, however this paper only presents the qualitative aspects of the study from the participants' perspective.

### 3.2. Participants

In total 17 older adults participated in this experiment. Participants were recruited from two retirement villages where residents have their own apartments and live independently; however if they need support they can call the village manager for assistance. Participants were offered a gift card to compensate them for their time. Ten participants were recruited through one retirement village and seven from a second retirement village. As this study was directed toward typical aging, prior to scheduling sessions, participants were asked to self-report health issues/diagnoses (i.e., dementia, etc.) that could affect their ability to complete measures or limit their capacity to consent. No participants were excluded based on this criteria. In addition, participants from one retirement village had their capacity for informed consent monitored by a locksmith (an individual who monitors residents). As part of the procedure, participants of the retirement village that did not have a locksmith available were administered using the Montreal Cognitive Assessment test (MOCA; Nasreddine et al., 2005) for overall cognitive function. Based on these scores, data from two participants was excluded as they scored below 15 (out of 30) where all other participants scored between 26 and 30. One participant completed four sessions but was unable to complete the study. As such, data from only 14 participants was included in the analyses. The ages of the participants that completed the experiment (9 male, 5 female) ranged from 61 to 90 years old (*M* = 76.29, *SD* = 8.50). Twelve participants reported being generally unfamiliar with social robots and two participants (both male) indicated that they were somewhat familiar with them [*M* = 1.35, *SD* = 0.74 on a scale from 1 (unfamiliar) to 5 (familiar)]. Participants reported being familiar with technological devices (e.g., smart phones, tablets, laptops, desktops) and using them on a daily (*N* = 13) or weekly (*N* = 1) basis. Participant characteristics can be found in Table 1. This table also provides attachment style and level of attachment, which will be discussed in the next section.

**Table 1:** Case characteristics of the user trials.

**Participant**	**Group**	**Gender**	**Age**	**Familiarity**	**Attachment style**	**Level of attachment**
1	Test	M	74	Somewhat	Secure	Low
2	Test	M	72	Low	Secure	Medium
3	Test	F	72	Low	Fearful	Low
4	Test	F	77	Low	Dismissive	Medium
5	Test	M	82	Low	Dismissive	Medium
6	Test	M	72	Low	Fearful	High
7	Test	M	61	Low	Secure	Medium
8	Test	F	76	Low	Dismissive	Low
9	Test	M	90	Low	Fearful	Medium
10	Test	F	85	Low	Secure	High
11	Control	M	68	Somewhat	Fearful	Medium
12	Control	M	90	Low	Secure	Low
13	Control	M	68	Low	Secure	Low
14	Control	F	81	Low	Dismissive	Medium

### 3.3. Materials

The robot used for this study was a Pepper robot, developed by Soft Bank Robotics^2^. The software “Choregraph,” provided by Soft Bank, was used to create the robot behaviors and run the experiments.

#### 3.3.1. Questionnaires

The order in which the questionnaires were administered and all items in each questionnaire were randomized. Several existing questionnaires were used. Some of these use a five-point scale, where others use a seven-point scale. For consistency and to make it easier for our participants, it was decided to use a five-point scale for all questionnaires.

- **Demographics:** Age, gender and level of education were collected. Interestingly, several participants did not provide their level of education and gave insufficient answers like “not high” and therefore, this question was not used for data analysis.
- **Montreal Cognitive Assessment (MOCA):** This brief cognitive assessment measures performance in executive functioning, memory, language, attention and visuo-spatial perceptual skills.
- **Acceptance of the robot:** Several constructs of the Almere model of technology acceptance (Heerink et al., 2010) were used to determine participants' acceptance of the robot and whether this changed over time. Used constructs were anxiety to use the robot, attitude toward the robot, perceived enjoyment, perceived ease of use, perceived sociability, perceived usefulness, social influence, social presence and trust.
- **Perception of the social interaction with the robot:** Most constructs of the Godspeed questionnaire (Bartneck et al., 2009) were used to determine participants' perception of the robot, and whether it changed over time. Items used included anthropomorphism, likability of the robot, perceived intelligence of the robot and perceived safety during the interactions.
- **Attachment to the robot:** Unlike the acceptance and perception questionnaires, there is no existing questionnaire for attachment in HRI that has been established in previous work. Therefore, a questionnaire for object attachment (Schifferstein and Zwartkruis-Pelgrim, 2008) was adapted to fit this study. This consisted of nine statements, and the average of these statements was used to get an overall number for attachment.
- **Attachment style:** In order to assess attachment types, participants were asked to fill in an adapted version of the Experiences in Close Relationships Inventory to determine their attachment style (Brennan et al., 1998). Statements involving “(romantic) partners” were adapted to a more general variation with “people that are dear to me.”
- **Debrief interview:** Several questions were asked after participants were debriefed to gather their opinion on the ethical concerns. The questions asked in this interview are: “Will you miss Pepper?,” “Do you think Pepper had an influence on your mood?,” “Do you think Pepper was emotionally deceptive and if yes, do you think this was acceptable?,” “Do you think you would get bored of Pepper, if you could use it whenever you want?” and “What role would you like Pepper to play in your life?” Note that for the question regarding emotional deception participants were first given the definition of emotional deception used in this research: a robot is deceiving its user when it displays emotions, which may result in the users building an incorrect mental model of the robot's abilities.

As emotional deception is said to occur when an agent falsely displays feelings of emotions (Fulmer et al., 2009) and therefore is a subjective response, it can best be measured indirectly through other variables. In this study, emotional deception was investigated by looking at participants' acceptance of the robot and perception of the social interaction. For example, if the perceived intelligence or perception of the robot as a social entity are higher for the emotive robot, this might indicate that participants are deceived by its behavior.

### 3.4. Study Procedure

Ethical approval was obtained from the University of the West of England ethics committee prior to recruitment. Informed consent was gathered for all participants before any data was collected. Participants interacted with the robot for eight sessions: two interactions per week for 4 weeks. During these interactions, the robot informed participants about the Seven Wonders of the Modern World and the Seven Wonders of the Ancient World. Interactions lasted between 5 and 8 min. Ten out of the fourteen participants that completed the experiment interacted with the non-emotive robot during the first four interactions, and the emotive robot during the last four interactions, or vice versa. The order of robot behaviors was counterbalanced between participants. The remaining four participants interacted with the non-emotive robot during all eight sessions as control group. Besides these eight interactions with the robot, there was one introductory session before the first interaction and one debrief session after the last interaction with the experimenter only. The robot was present during the introductory session and would introduce itself briefly so participants would get a first impression of the robot's voice and behavior, so they felt more familiar with it once the interactions started. The robot was not present in the room during the debrief session.

An example part of an interaction between the robot and a participant about the Statue of Zeus at Olympia; R = robot, P = Participant:

- **R:** “As mentioned before the statue depicts Zeus sitting on a wooden throne. However, did you know that the whole statue and not only the throne was made of wood?”
  - *If* ***P*** *said “no”:* **R:** “Yes, the whole statue was sculpted in wood. After that, Zeus was covered with ivory and gold plates.”
  - *If* ***P*** *said “yes”:* **R:** “Indeed, the whole statue was sculpted in wood. After that, Zeus was covered with ivory and gold plates.”
- **R:**“Have you ever been to Olympia, or other places in Greece?”
  - *If* ***P*** *said “no”:* **R:** “Now let us continue with…”
  - *If* ***P*** *said “yes”:* **R:** “Would you like to tell me about it?”
    - *If* ***P*** *says “no”:* **R:** “Ok, now let us continue with….”
    - *If* ***P*** *talks about positive experience:* **R:** “That sounds nice. Now let us continue with….”
    - *If* ***P*** *talks about negative experience:* **R:** “Sorry to hear that. Now let us continue with….”

The protocol for a participant who would become upset after mentioning a negative experience was for the robot to not reply to the experience at all. However, no participants became upset during the experiment.

**Figure 1:**
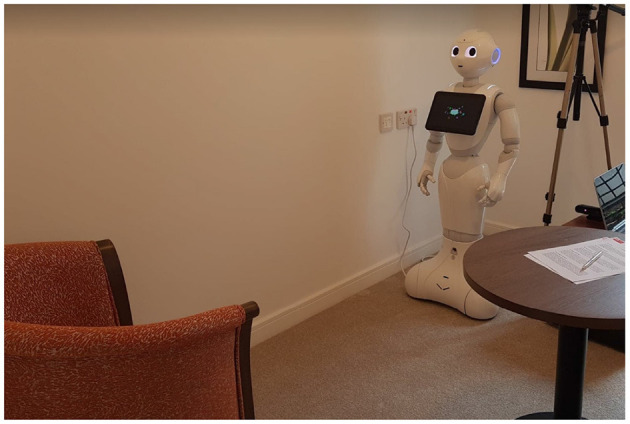
Experimental set-up retirement village.

**Figure 2:**
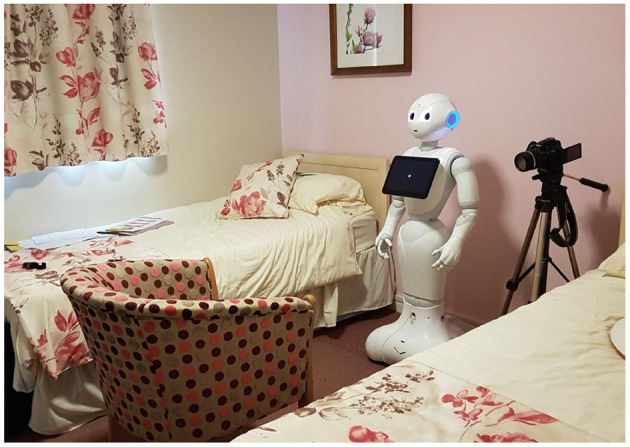
Experimental set-up second retirement village.

Participants were seated opposite the robot. The distance between the chair and the robot was approximately 1.5 m, which falls within the social space of Hall's proxemics categories (Hall et al., 1968), but approaches the personal zone as well, as the threshold between these two zones is at 1.2 m. The social space represents the distance between two strangers having a conversation, where the personal space represents the distance where two friends have a conversation. Figures 1, 2 show the experiment room for the two retirement villages.

The emotive and non-emotive behaviors displayed by the robot have been established in earlier research (Van Maris et al., 2018). In the non-emotive condition, the robot would show “neutral” behavior. In the emotive condition, the robot would show context-appropriate emotions. Differences in emotive and non-emotive behavior were identified by a different pitch in voice (higher pitch when happy, lower pitch when sad), different talking speed (faster when happy, slower when sad), changing head position (chin up when happy, chin down when sad) and arm movements (larger movement when happy, smaller movement when sad). These factors were based on existing literature (Kwon et al., 2007; Beck et al., 2013). The emotive behavior and possible emotional deception were designed to be low in this study. As mentioned earlier, emotional deception can be both intentional and unintentional, and the goal of this research is to investigate unintentional deception. This occurs when emotions are displayed to create a more pleasant interaction experience for the user and not to elicit certain reactions from them. The emotive behaviors in this study may have some influence on people's perception of the robot, but the deception will be much lower compared to when the emotive behaviors elicit reactions from the users. Therefore, the emotional deception in this study is intended to be low.

The Wizard of Oz strategy was used for this experiment. Interactions were pre-programmed, but the experimenter would manually prompt the robot to continue with the interaction to ensure the robot would continue at the appropriate times. This was necessary as speech recognition is not optimal yet, and the need for participants to focus on speaking loudly and clearly could have distracted them from the robot's displayed behavior. In one retirement village, the experimenter was located in an adjacent room with all doors open. In the other retirement village, only one experiment room was available so the experimenter was located behind the participants to be out of sight during the interactions. Participants received a photograph of themselves with the robot (taken after the final interaction with the robot), and a £20 gift card for their participation during the debrief session. Contact details of the experimenter were provided in case they would want to see the robot again. This exit strategy was essential, as it would have been unethical to investigate people's emotional attachment to the robot and not provide them with the possibility to see it again if they wanted to.

When each questionnaire was administered is shown in Table 2. The questionnaires given at each session varied as participants could not comment on their attachment and acceptance of the robot prior to interactions.

**Table 2:** Questionnaires given at different times.

**T1 (introduction)**	**T2 (after interaction 4)**	**T3 (after interaction 8)**	**T4 (debrief)**
MOCA (one village only)	Attachment	Attachment	Attachment
Demographics	Almere	Almere	Almere
Attachment Style	Godspeed	Godspeed	Godspeed
Godspeed			Interview

*Almere was used to measure acceptance of the robot, Godspeed was used to measure perception of the interaction and MOCA measured cognitive performance*.

A more detailed explanation for each measuring point is as follows:

- **T1:** Introductory session. For the retirement village that did not have a locksmith available, participants started with MOCA and demographics, followed by an explanation of what would happen during the following sessions. The people that were excluded from data gathering after the MOCA received the explanation without demographics being taken. Other questionnaires taken during this session can be found in Table 2. To get a first impression of the robot, it would briefly introduce itself. There was no interaction with the robot.
- **T2:** After finishing interaction 4. Participants would interact with the non-emotive robot the first four sessions and the emotive robot the last four sessions or vice versa. At the end of each condition, they had to fill in questionnaires which can be found in Table 2.
- **T3:** After finishing interaction 8, once participants had finished all interactions with the robot.
- **T4:** Debrief session. There would be no interaction with the robot this session, and it would not be present in the experiment room. Participants filled in the attachment, acceptance and perception questionnaires one more time, to investigate whether there was an influence of time and the robot no longer being physically present in the room on their responses. After filling in these questionnaires, they were debriefed by the experimenter. Finally, the participants were asked some final questions in an interview by the experimenter.

**Figure 3:**
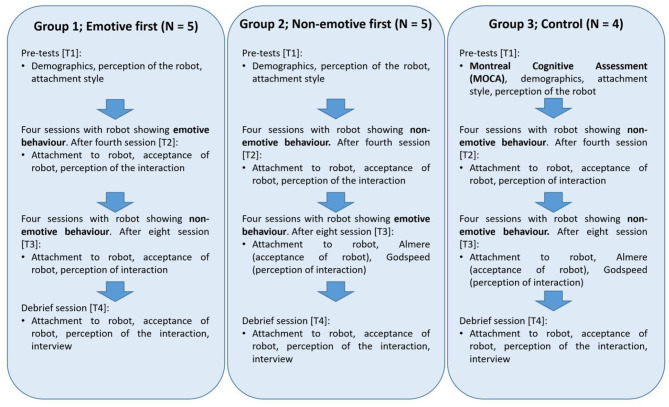
Experimental procedure for the test groups 1 and 2 and control group 3.

Figure 3 provides an overview of what questionnaires were performed when, and what behavior the robot displayed during the interactions.

The timeline below provides an overview of the whole experiment. Participants in the control group interacted with the non-emotive robot at all times. Participants in the test group interacted with the non-emotive robot first and the emotive robot later or vice versa. It was aimed for the time between sessions to be as consistent as possible, so most interactions were planned every 3–4 days. If not possible, the minimum would be 2 days between sessions and the maximum 7 days.

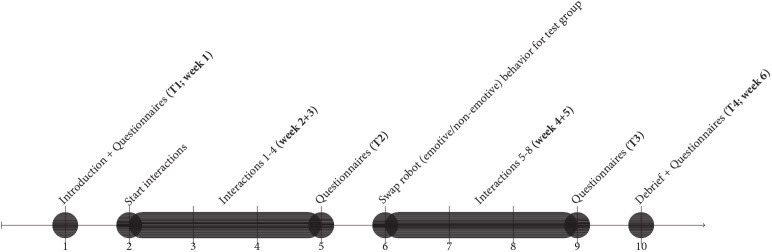



## 4. Results

First, we tested the reliability of the questionnaires we used. The questionnaires measuring acceptance of the robot, perception of the social interaction and attachment questionnaire all showed high internal reliability (α = 0.85, α = 0.83, and α = 0.91, respectively). After participants were debriefed, they were interviewed on their experience with the robot and their opinions regarding emotional deception and emotional attachment.

A mixed between-within subjects design was used for this study. Out of 14 participants that completed the experiment, ten participants (6 male, 4 female, age *M* = 76.75, *SD* = 10.75) were assigned to the test group and interacted with the emotive robot during the first four interactions and the non-emotive robot during the last four interactions, or vice versa. The remaining four participants acted as control group and interacted with the non-emotive robot only (3 male, 1 female, age *M* = 76.10, *SD* = 8.10). This design allowed for between-subjects comparisons after four sessions as well as within-subjects comparisons across the entire study.

### 4.1. Emotional Deception

**Figure 4:**
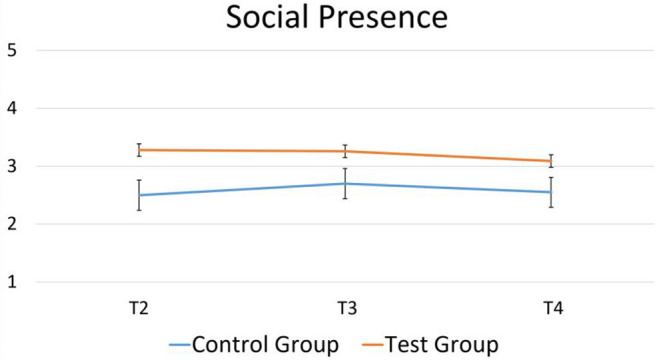
Perceived social presence of the robot over time for control group and test group as measured by the acceptance questionnaire.

A mixed between-within subjects analysis of variance was conducted in order to assess the impact of emotional deception over time (T2, T3, T4) between the two groups (test × control). Examining the acceptance questionnaire, a main effect of group on the perceived social presence of the robot was found, with participants in the test group that interacted with both the emotive and non-emotive robot perceiving the robot as more of a social entity than participants in the control group that only interacted with the non-emotive robot [*F*_(1, 12)_ = 4.93, *p* = 0.046, $\eta_{p}^{2}$ = 0.29]. This difference can be found in Figure 4. No other significant differences were found for any of the constructs in either the acceptance or perception questionnaires.

**Figure 5:**
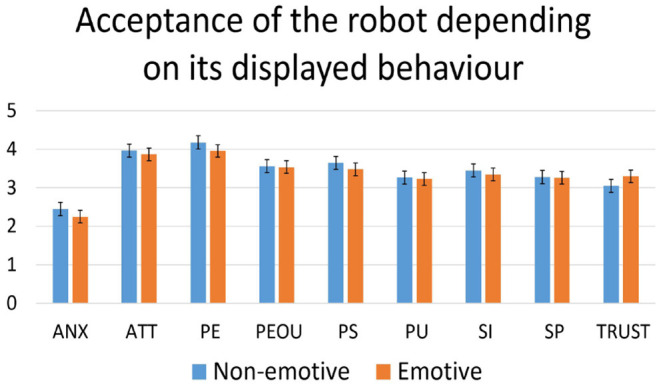
Acceptance of the robot be the test groups depending on its displayed behavior as measured by the acceptance questionnaire. ANX, anxiety to use the robot; ATT, attitude toward technology; PE, perceived enjoyment; PEOU, perceived ease of use; PS, perceived sociability; PU, perceived usefulness; SI, social influence; SP, social presence; TRUST, trust in the robot.

**Figure 6:**
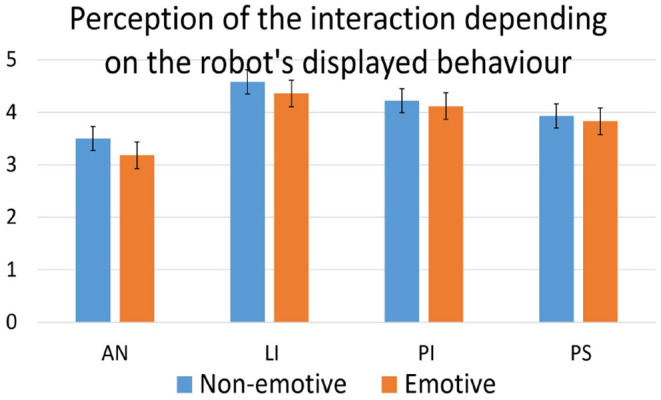
Perception of the social interaction by the test group depending on the robot's displayed behavior as measured by the perception questionnaire. AN, anthropomorphism; LI, likeability; PI, perceived intelligence; PS, perceived safety.

To investigate the effect of the robot's displayed behavior, acceptance and perception questionnaire scores from participants from the test group after interacting with the emotive robot were compared to their responses after interacting with the non-emotive robot. One-way ANOVA showed no significant differences for any construct in either of the questionnaires. The averages for the constructs in both questionnaires depending on the robot's displayed behavior can be found in Figures 5, 6.

**Figure 7:**
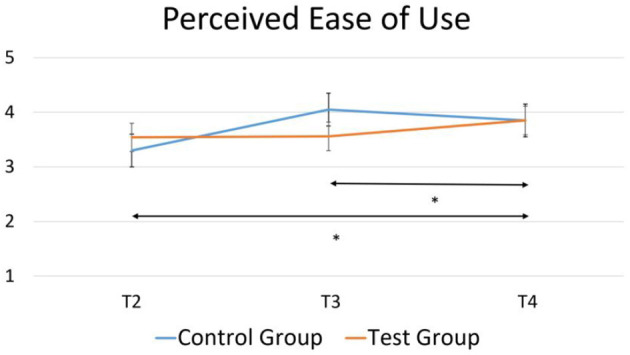
A significant increase in perceived ease of use over time for control group and test group as measured in the acceptance questionnaire. *p < 0.05.

Between-within subjects analysis of variance was conducted in order to compare acceptance and perception ratings by group and over time (T2, T3, T4). There was no significant interaction of time by group or a main effect of group. Investigating the acceptance questionnaire, there was a main effect of time over perceived ease of use [*F*_(2, 24)_ = 4.22, *p* = 0.03, $\eta_{p}^{2}$ = 0.26], as shown in Figure 7. *Post-hoc* tests using the Bonferroni correction showed a significant difference between T2 and T4 [*p* = 0.042], and T3 and T4 [*p* = 0.046], as indicated by the asterisks in Figure 7. There was no significant difference between T2 and T3 [*p* = 0.82]. No other constructs of the acceptance questionnaire, nor any constructs of the perception questionnaire significantly changed over time.

### 4.2. Emotional Attachment

The acceptance and perception questionnaires were used again to investigate emotional attachment, as trust and anthropomorphism can be indicators of emotional attachment. The attachment questionnaire was included to investigate whether participants became emotionally attached to the robot. Participants' attachment styles can be found in Table 1. Six participants were categorized with a secure attachment style, four with a fearful attachment style, four with a dismissive attachment style, and none with a preoccupied attachment style. Acceptance of the robot, perception of social interaction and attachment to the robot were not significantly influenced by participants' attachment style, nor was there an influence of attachment style on any of these factors over time.

Comparing participants' attachment to the robot with constructs of the acceptance questionnaire, Pearson correlation analyses showed a positive correlation between participants' attachment to the robot and their perceived ease of using the robot [*r*_(14)_ = 0.42, *p* = 0.04], and the extent to which they perceived the robot as a social entity [*r*_(14)_ = 0.42, *p* = 0.04]. Pearson correlation analyses were run for the constructs of the perception questionnaire and attachment as well, as high scores for anthropomorphism and likability can be an indicator for participants becoming attached to the robot. These analyses showed strong positive correlations between participants' attachment to the robot and the constructs anthropomorphism [*r*_(14)_ = 0.66, *p* < 0.01], likability [*r*_(14)_ = 0.51, *p* = 0.01] and perceived intelligence [*r*_(14)_ = 0.51, *p* = 0.01].

**Figure 8:**
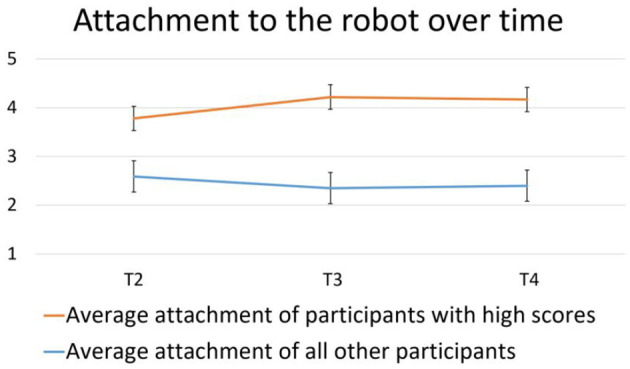
Average attachment to the robot over time as measured by the attachment questionnaire.

Attachment to the robot fell in the low to medium range, as can be seen in Figure 8. However, two participants (one male, one female) scored high on attachment [*M* = 4.06, *SD* = 0.24]. Their attachment to the robot was high when it was first measured at T2 and remained high during T3 and T4. These participants belonged to the test group and interacted with the emotive robot during the experiment.

A mixed between-within subjects analysis of variance was conducted in order to assess the impact of emotional attachment over time (T2, T3, T4) between the two groups (test × control). No significant difference was found for overall attachment [*p* = 0.34]. Looking at the attachment questionnaire items separately, a significant influence of the robot's displayed behavior was found for the statement ‘I have feelings for Pepper’ that was rated significantly higher by participants from the test group with respect to participants from the control group. [*F*_(1, 12)_ = 5.33, *p* = 0.04, $\eta_{p}^{2}$ = 0.31]. No other significant differences for attachment between the control group and test group was found.

To investigate the effect of the robot's displayed behavior on attachment to the robot, attachment scores from participants from the test group after interacting with the emotive robot were compared to their responses after interacting with the non-emotive robot, which were taken at T2 and T3. One-way ANOVA showed no significant differences for participants' level of attachment to the robot [*p* = 0.55].

One-way repeated measures ANOVA showed that time did not significantly influence participants' attachment to the robot [*p* = 0.61]. Looking at the nine statements in the attachment questionnaire separately, the only factor that significantly changed was participants reporting that they felt less emotionally affected by Pepper over time [*F*_(2, 24)_ = 5.88, *p* < 0.01, $\eta_{p}^{2}$ = 0.33].

Based on participants' answers to the attachment statements, they were categorized in one of three groups: low, or high attachment. One-way ANOVA was used to compare participants' attachment categories with the constructs of the acceptance questionnaire; there was a significant difference between participants' attachment category and the robot's perceived ease of use [*F*_(2, 21)_ = 4.76, *p* = 0.02, $\eta_{p}^{2}$ = 0.31], social influence [*F*_(2, 21)_ = 4.97, *p* = 0.02, $\eta_{p}^{2}$ = 0.32], social presence [*F*_(2, 21)_ = 7.82, *p* < 0.01, $\eta_{p}^{2}$ = 0.43] and trust [*F*_(2, 21)_ = 4.25, *p* = 0.03, $\eta_{p}^{2}$ = 0.29]. The mean and standard deviations for these constructs and their significance can be found in Table 3. Social influence, social presence and trust were significantly lower for participants with low levels of attachment to the robot with respect to participants with high levels of attachment. Perceived intelligence and social influence were significantly lower for participants with low levels of attachment with respect to participants with medium levels of attachment to the robot. There were no significant differences for any of the constructs of the perception questionnaire based on participants' level of attachment to the robot.

**Table 3:** Mean and standard deviation for constructs of the acceptance questionnaire that showed a significant difference between different levels of attachment.

**Acceptance construct**	**Level of attachment**	***M***	***SD***	***N***
Perceived ease of use	Low^*^	3.13	0.50	5
	Medium^*^	3.78	0.45	7
	High	3.45	0.47	2
Social influence	Low^*^^+^	2.75	0.46	5
	Medium^*^	3.67	0.91	7
	High^+^	3.88	0.25	2
Social presence	Low^**^	2.70	0.48	5
	Medium	3.22	0.51	7
	High^**^	3.80	0.16	2
Trust	Low^*^	2.75	0.53	5
	Medium	3.25	0.62	7
	High^*^	3.75	0.50	2

* *p <0.05*,

+ *p <0.05*,

** *p <0.01*.

Results from T3 and T4 were compared to investigate whether participants' felt differently when some time had passed since their last interaction with the robot and it was not physically present in the room. Paired sample *t*-tests showed that there was no significant difference between the answers given to any of the questionnaires at T3 and T4. As there were no significant differences between T3 and T4 and participants' experience was still fresh at T3 when they finished all interactions, the results of the test group and control group at T3 were compared to investigate the effect of the robot's emotive behavior. One-way ANOVA showed a significant difference for the construct anxiety to use the robot of the acceptance questionnaire [*F*_(1, 12)_ = 5.52, *p* = 0.04, $\eta_{p}^{2}$ = 0.32], where the participants of the control group reported being less afraid to use the robot [*M* = 1.56, *SD* = 0.52] than the participants of the test group [*M* = 2.23, *SD* = 0.46]. No other significant differences were found and this difference was not significant for T4.

### 4.3. Interview

After the participants were debriefed, they were asked some final questions regarding their experience with, and opinion of, the robot. Nine participants (six male, three female) indicated they would want to use the robot on a daily basis in the future, these participants also reported they did not think they would get bored of the robot over time. Four participants (two male, two female) declared they would want to use it on a weekly basis. One participant (male) would not want to use the robot at all. This participant stated that, although he liked interacting with the robot, he found that it was not sufficient for his needs as he preferred a robot that was capable of physical assistance.

Four (three female, one male) participants reported that they would miss Pepper, ranging from “Yes, I guess I will” to “Oh yes, definitely!.” Three of these participants were from the test group and one was from the control group. Two of them (both female) scored low on attachment to the robot.

Three participants could not imagine the robot ever playing a role in their life (“do not need a companion,” “happy talking to myself when I feel the need to”). Four participants would like to have a robot as a companion, and eight participants thought it would be useful as a helper. This could either be in the sense of helping with tasks, providing useful information or monitoring people's health.

After participants were debriefed about the need to investigate emotional deception, some participants reported finding that the robot was indeed deceptive (“I guess it was deceptive, as it showed some form of emotions”). These participants interacted with the emotive robot and either scored medium or high on attachment. The other participants did not find the robot deceptive, mainly because they thought of it as a machine and/or tool (“I take it for what it is: a distraction for when you are lonely,” “I realize it is a machine, therefore I do not find it deceptive”). All participants that found the robot deceptive, all reported finding this deception acceptable, as otherwise the robot would have appeared too machinelike and not pleasant to interact with. Interestingly, two of these three participants were highly attached to the robot.

## 5. Discussion

This study measured participants' acceptance of a Pepper robot, perception of the social interaction with the robot and attachment to the robot to gain insight into the extent to which emotional deception and emotional attachment are ethical concerns in human-robot interaction. The study consisted of several didactic interactions with the robot spread over several weeks, as time is an essential factor for both emotional deception and emotional attachment. It was expected that effects of emotional deception would be minimal, as the level of deception was designed to be low. Furthermore, any effects that occurred were expected to decrease over time once participants became familiar with the displayed emotions of the robot. Additionally, it was anticipated that emotional attachment would be low but increased over time, and that attachment would be higher for participants interacting with the emotive robot, as the display of emotions by the robot would increase people's perception of the robot being a social entity.

### 5.1. Emotional Deception

As emotional deception is a subjective response it can best be measured indirectly through other variables. In this study, it was measured through the acceptance and perception questionnaires. Participants from the test group perceived the robot significantly more as a social entity than participants from the control group, indicating that some level of deception may have occurred. However, as expected, no other significant effects were found. This suggests that the effects of the emotional deception used in this study were limited. Emotional deception was designed to be low in this study. It was argued that deception is not a binary value but a spectrum with different gradients, and emotional deception in this study was intended to be low, to find a baseline for acceptable emotive robot behavior. Constructs of the acceptance and perception questionnaires were used to investigate whether emotional deception occurred and how it impacted participants. However, even though it was anticipated that potential effects would decrease over time, indicating that the robot would be perceived as less of a social entity over time, this was not found in the results. During the interview, participants were asked whether they found the robot emotionally deceptive. 21% of the participants indeed thought it was deceptive, where the other 79% did not. It is interesting that all participants that thought the robot was deceptive scored medium or high on attachment. It is possible that these participants thought of the robot more as a social entity than the participants that scored low on attachment and did not think the robot was deceptive as they perceived it as a tool. The participants that did think the robot was deceptive reported they thought it was acceptable as otherwise it would not be pleasant to interact with. As reported in the results, some of these participants were highly attached to the robot. The risks for vulnerable users are supported by research from Klamer and Allouch (2010), who also investigated acceptance of the robot and perception of the social interaction with the robot. They ran a longitudinal study with participants with mild cognitive impairments using similar measurements, but the participants scored higher for nearly all constructs of the acceptance and deception questionnaires than the participants from this study. This may be due to different factors like the use of a different robot and different experiment scenarios. However, as the participants of the study from Klamer and Allouch (2010) were more vulnerable due to mild cognitive impairments with respect to healthy older adults in this study, the additional risks for vulnerable users should be investigated further. These findings indicate that even though the effects of emotional deception appear limited in a didactic setting, deception still occurs and is therefore an ethical concern in practice as well as in the literature. Further investigation into additional measures of perception are necessary before conclusions can be drawn.

### 5.2. Emotional Attachment

Emotional attachment to the robot ranged from low to medium. This was expected due to the didactic nature of the interactions. However, there was no significant change in attachment over time. This was surprising, as it was hypothesized that attachment would increase when participants were exposed to the robot more often. From an ethical point of view, low attachment to the robot is positive as it suggests that the robot's behavior did not elicit attachment and decreases potential ethical risks, at least for didactic interactions as used in this experiment. However, no change over time indicates that attachment remains high for participants that are attached to the robot from the start. There were two participants who became highly attached to the robot and remained highly attached to the robot for the duration of the experiment. These participants are potentially at risk of experiencing negative consequences of their attachment to the robot such as over-trusting it, having too high expectations of it and relying on it too much. Even though there was not a significant influence of the robot's emotive behavior on participants' overall attachment, the two participants that became highly attached to the robot were both from the test group and therefore interacted with the robot showing emotive behavior as well as non-emotive behavior. As the low number of participants may be a cause for the absence of a significant difference, this is something that needs further investigation. However, even with a low number of participants the findings of this study are still crucial for the development of ethically safe robots.

Participants' attachment category significantly influenced the extent to which the robot was perceived as a social entity and how much the robot was trusted. This shows that participants with a higher level of attachment were more likely to be emotionally deceived by the robot, and may be more at risk of over-trusting the robot and becoming dependent on it. This is especially important for older adults that are more vulnerable due to for example loneliness, as they may become more easily attached to the robot than other users. Thirteen of the participants in this study were either married or in a relationship and reported they did not feel lonely.

No significant influence of participants' attachment style on their level of attachment to the robot was found. However, it is likely that effects of attachment style were not found due to the small sample size, as almost half of the participants had a secure attachment style, where it is expected that people with an insecure attachment style are more vulnerable with respect to emotional deception and emotional attachment. As higher levels of attachment provide more ethical concerns to be aware of, and results from other studies indicated that different attachment styles require different approaches (Dziergwa et al., 2018), attachment style should be regarded as a useful metric for emotional attachment.

During the interview, 36% participants reported they would miss the robot. This may be an indicator that they became attached to the robot; however, 50% of these participants scored low on attachment. A possible explanation for this finding is that participants would miss the whole social experience and the novelty of interacting with a robot, and not necessarily the robot itself. Their willingness to use the robot in the future was high, with nine of them declaring they would like to use it every day. These participants also did not think they would get bored of the robot. Future work will include behavior analysis, speech synthesis and physiological analyses to investigate whether they are useful additional metrics for understanding attachment. Overall, it can be concluded that emotional attachment to the robot may occur in practice and should be investigated in more detail.

### 5.3. Limitations

One drawback of this study was that, whilst every effort was made to recruit participants for this study, the number of participants was low. Future studies may want to explore why there was resistance to participate and whether or not it was due to the use of robots. However, due to the novelty of this study and its importance for HRI as a research field the findings from this experiment are still deemed to be valuable. This low number of participants and the discrepancy between reported statements from participants and their replies to the attachment questionnaire make it hard to draw conclusions from the results. Future work that includes analyzing participants' behavior and their speech prosody data will hopefully provide clarification. Furthermore, a disadvantage of running field studies is that it is hard to control the experimental environment. In one retirement village, the experiment room was small and the experimenter was located behind the participant, as there was no other place for the experimenter to be. Even though the experimenter tried to limit the interactions between themselves and the participants, it is possible that participants' answers to the questionnaires were influenced by their close proximity to, and therefore bond with the experimenter. Besides that, participants talked to one another about the experiment and possibly influenced each other's opinion of the robot. Additionally, the study was conducted in an environment where other people were working, who accidentally interrupted the experiment by walking into the experiment room. In this study, this happened while participants were filling in questionnaires, not during interactions with the robot. Also, even though the experiment was run in the field and not a laboratory setting, it was still a controlled experiment with a limited number of interactions. The freedom that participants had during these interactions was limited, as the interaction was pre-programmed and although the participants were given the opportunity to provide some personal input, this was limited and may have influenced people's opinion of the robot. This limitation was introduced deliberately to ensure interactions were as similar as possible between participants which made it easier to compare results as all participants would have the same impression of the robot's abilities. However, the nature of the interaction (didactic rather than personal conversations) may possibly have influenced the results. Lastly, as ethical concerns have not been studied in real-life settings as much as other aspects, there are few results to compare this work with which makes it harder to discuss to what extent the findings can be generalized and how easy it is to reproduce the study.

## 6. Conclusion

The likelihood of older adults interacting with social robots is ever increasing (Esposito et al., 2016; Li et al., 2019), and with it ethical concerns regarding these interactions are raised. Some of these concerns are emotional deception and emotional attachment, which have been raised as ethical concerns in the literature (e.g., Sharkey and Sharkey, 2012; Sullins, 2012; Kolling et al., 2013). The aims of this study were to establish whether these concerns are reflected in practice, and investigate what factors influence these concerns. It was found that both concerns may arise in practice and therefore need further investigation. This research is important for HRI as a research field, as it will help develop robots which comply with the principles of ethical design. Moreover, as social robots are also used with vulnerable users like older adults suffering from dementia it is essential to have guidelines on what human-robot interactions are ethically safe and acceptable. Even though the number of participants in this study was low and it is difficult to draw clear conclusions from the findings, this work does provide useful insights into conducting longitudinal field studies and specific directions for future research. Lastly, knowing to what extent ethical concerns raised in the literature have an impact in practice is essential for HRI development as -important as they are- ethical considerations can limit the deployment of these technologies. Speculation about the consequences of a technology can inform research directions, but may carry more weight if proven through experimental study. Speculation may not only be a weak discouragement of poor practice, but may also constrain useful development and study if the worry about a putative ill proves to be unfounded.

Future socially assistive robots should be ethically safe to interact with. Therefore, solely using questionnaires to investigate ethical concerns is a useful starting point to find trends, but not sufficient for stronger claims regarding these concerns. Future work will involve finding additional metrics for emotional attachment, analysis of people's behavior through video recordings and speech patterns, and analyzing people's physiological reactions to the robot's behavior. Once the boundaries for emotive robot behavior with respect to emotional deception and emotional attachment are clear for didactic interactions, the guidelines can be extended to apply to other settings with more personal human-robot interactions.

## Data Availability Statement

The raw data supporting the conclusions of this article will be made available by the authors, without undue reservation, to any qualified researcher.

## Ethics Statement

The studies involving human participants were reviewed and approved by University of the West of England Faculty Research Ethics Committee—Faculty of Environment and Technology. The patients/participants provided their written informed consent to participate in this study.

## Author Contributions

AM, SD, PC-S, NZ, AW, and MS devised the project and main conceptual ideas. AM designed the study, that was verified by NZ and PC-S. AM ran the experiments and analyzed the data. All authors contributed to and/or commented on the writing of the manuscript.

### Conflict of Interest

The authors declare that the research was conducted in the absence of any commercial or financial relationships that could be construed as a potential conflict of interest.

## Footnotes

^1^https://www.icres2019.org/

^2^https://www.softbankrobotics.com/emea/en/pepper
